# Potential Bioactivities, Chemical Composition, and Conformation Studies of Exopolysaccharide-Derived *Aspergillus* sp. Strain GAD7

**DOI:** 10.3390/jof10090659

**Published:** 2024-09-19

**Authors:** Mohamed I. A. Ibrahim, Hassan A. H. Ibrahim, Tatsuki Haga, Atsuhiko Ishida, Tatsuo Nehira, Koichi Matsuo, Ahmed M. Gad

**Affiliations:** 1 Research Institute for Synchrotron Radiation Science, HiSOR, Hiroshima University, Higashi-Hiroshima 739-0046, Hiroshima, Japan; m235936@hiroshima-u.ac.jp; 2 National Institute of Oceanography and Fisheries (NIOF), Cairo 4262110, Egypt; drhassan1973@yahoo.com; 3 Laboratory of Molecular Brain Science, Graduate School of Integrated Sciences for Life, Hiroshima University, Higashi-Hiroshima 739-8521, Hiroshima, Japan; aishida@hiroshima-u.ac.jp; 4 Graduate School of Integrated Sciences for Life, Hiroshima University, Higashi-Hiroshima 739-8521, Hiroshima, Japan; 5 International Institute for Sustainability with Knotted Chiral Meta Matter (WPI-SKCM2), Hiroshima University, Higashi-Hiroshima 739-8526, Hiroshima, Japan; 6 Research Institute for Semiconductor Engineering, Hiroshima University, Higashi-Hiroshima 739-8527, Hiroshima, Japan

**Keywords:** exopolysaccharide, *Aspergillus* sp., structural analysis, helix-like conformation, circular dichroism, biological activities

## Abstract

This research identified a marine fungal isolate, *Aspergillus* sp. strain GAD7, which produces an acidic and sulfated extracellular polysaccharide (EPS) with notable anticoagulant and antioxidant properties. Six fungal strains from the Egyptian Mediterranean Sea were screened for EPS production, with *Aspergillus* sp. strain GAD7 (EPS-AG7) being the most potent, yielding ~5.19 ± 0.017 g/L. EPS-AG7 was characterized using UV-Vis and FTIR analyses, revealing high carbohydrate (87.5%) and sulfate (24%) contents. HPLC and GC-MS analyses determined that EPS-AG7 is a heterogeneous acidic polysaccharide with an average molecular weight $\left(\bar{Mw}\right)$ of ~7.34 × 10^3^ Da, composed of mannose, glucose, arabinose, galacturonic acid, galactose, and lyxose in a molar ratio of 6.6:3.9:1.8:1.3:1.1:1.0, linked through α- and β-glycosidic linkages as confirmed by NMR analysis. EPS-AG7 adopted a triple helix-like conformation, as evidenced by UV-Vis (Congo Red experiment) and circular dichroism (CD) studies. This helical arrangement demonstrated stability under various experimental conditions, including concentration, ionic strength, temperature, and lipid interactions. EPS-AG7 exhibited significant anticoagulant activity, doubling blood coagulation time at a concentration of 3.0 mg/mL, and showed significant antioxidant activity, with scavenging activities reaching up to 85.90% and 58.64% in DPPH and ABTS^+^ assays at 5.0 mg/mL, and EC50 values of 1.40 mg/mL and 3.80 mg/mL, respectively. These findings highlight the potential of EPS-AG7 for therapeutic applications due to its potent biological activities.

## 1. Introduction

Polysaccharides are widely distributed in nature and can originate from various origins such as seaweed, plants, and microorganisms like bacteria and fungi. Polysaccharides derived from microorganisms exhibit distinct molecular structures and conformations, offering promising properties for diverse applications [1,2,3]. Fungi have emerged as a significant group of microorganisms due to their abundance in producing natural compounds and active metabolites [4,5,6]. They can be isolated from different environments, including terrestrial and marine substrates [7]. Coastal sediments, known for their higher nutrient content compared to seawater, harbor a greater diversity of fungi [8]. Despite this, global research on fungi, especially those from marine environments, remains limited. Marine-derived fungi contribute significantly to microbial natural products, accounting for approximately 64% of these resources between 2010 and 2013 [9].

Fungal polysaccharides have received considerable attention among natural products because of their biodegradability and biocompatibility [1,3]. They are increasingly recognized as viable alternatives to polysaccharides derived from seaweed and plants and can be synthesized at various stages of fungal life cycle to serve diverse purposes [10]. Fungal polysaccharides are categorized into extracellular, cell-walled, and intracellular types [11], playing crucial roles in cellular recognition and provide protection against phagocytosis, osmotic stress, phage attack, toxic substances, and predation by protozoa [12]. Among these, extracellular polysaccharides (EPS) are favored due to their high production yield, as well as straightforward separation and purification procedures compared to intracellular and cell wall polysaccharides [13]. In recent decades, EPS, particularly from marine fungi, has attracted significant research interest in biomedical applications due to their potential antioxidant, anti-inflammatory, antiviral, anticoagulant, wound dressing, immunomodulatory, and anticancer properties [1,3,14,15]. Additionally, EPS find industrial use in the food sector as gelling agents, thickeners, stabilizers, emulsifiers, and bioadhesives [16]. Structurally, EPS are composed of monosaccharide moieties linked through glycosidic linkages. They are classified into homopolysaccharides consisting of a single type of sugar and heteropolysaccharides composed of two or more types of sugar units [17]. The properties and functions of polysaccharides are influenced by several issues such as the composition and sequence of sugar units, degree of branching, type of glycosidic bonds, molecular weight, and conformation, collectively described by the term “Structure–Activity Relationship (SAR)” [1,2,3]. Understanding of the SAR for polysaccharides remains limited due to insufficient data associated with their structural complexity. Several methods including FTIR, NMR, HPLC, GC-MS, UV-Vis, SAXS, and SEM are employed to characterize polysaccharide structures, describing sugar composition, sequence and type of linkages between sugar moieties, degree of branching, crystalline/amorphous ratio, and textural morphology, while circular dichroism (CD) technique is utilized to examine the structuration and conformation of polysaccharides [18,19,20].

The SAR of fungal EPS underscores how structural features dictate their biological activities and potential applications, highlighting the importance of detailed structural characterization for functional application development. For example, *Aspergillus fumigatus* produces a linear heterogeneous polysaccharide composed of galactose and N-acetylgalactosamine linked through α1-4 bonds, demonstrating immunosuppressive activity [21]. Similarly, different EPS produced by *Aspergillus terreus* strains exhibit varied functionalities: one type, primarily composed of O-glucosamine with traces of D-fucose, D-glucose, and D-galactose, shows anticoagulant activity and cytotoxic effects against breast cancer and human skin fibroblasts [12]. Another EPS from the same species, composed of mannose, galactose, and glucose in a 40.5:35.2:24.3 molar ratio, displays immunomodulatory effects by inducing production of nitric oxide, superoxide anion, interleukin 6, and TNF-α in murine macrophages [22]. Polysaccharides from various fungi highlight their diverse biological activities based on structural variations. For example, *Agaricus bisporus* polysaccharides, primarily mannogalactan, enhance enzyme synthesis and promote pro-inflammatory cytokines, whereas polysaccharides from *Agaricus blazei*, rich in β-glucan, reduce cytokine production under lipopolysaccharide stimulation [23]. *Ganoderma sinense* and *Ganoderma lucidum* produce polysaccharides with molecular weights $(\bar{Mw)}$ ranging from 13.4 × 10^3^ to 17.0 × 10^3^ Da, composed of mannose, glucose, and galactose linked by α- and β-linkages, exhibiting tumor-suppressive activity and macrophage activation [24]. Additionally, fucose-containing acidic polysaccharides from *Agaricus blazei* with $(\bar{Mw)}$ of 3.5 × 10^5^ Da and composed of l-fucose, l-arabinose, d-galactose, d-xylose, and d-galacturonic acid showed reduced TNF-α stimulation upon removal of terminal L-fucosyl residues [25]. Polysaccharides from *Laminaria japonica*, rich in mannose, galactose, fucose, and glucuronic acid, demonstrate potent anticoagulant activities attributed to their high uronic acid and sulfate contents. Additionally, polysaccharides with triple helical conformation exhibit significant anti-tumor and immune-supporting bioactivities [26,27].

The novelty of the current study lies in its comprehensive characterization and optimization of EPS from the marine fungus *Aspergillus* sp. strain GAD7, employing integrated spectroscopic techniques. This study includes genetic identification, production, purification, and detailed structural characterization using NMR, FTIR, HPLC, and size exclusion chromatography (SEC). It uniquely investigates the polysaccharide’s conformation through UV-Vis (Congo Red experiment) and CD technique, examining the factors (i.e., concentration, temperature, ionic effect, and lipid interaction) influencing this conformation. Additionally, the study evaluates the EPS’s antioxidant and anticoagulant activities, providing rare insights into its potential therapeutic applications.

## 2. Material and Methods

### 2.1. Samples Collection and Marine Fungi Isolation

**Figure 1:**
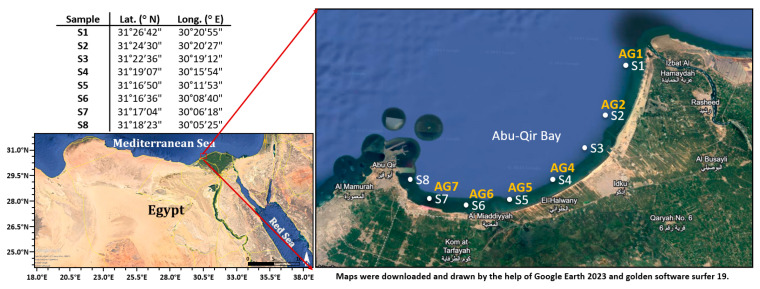
Sampling sites for marine algae samples and the corresponding fungi isolates along the Abu-Qir shore in Egypt during September 2022.

Eight marine algae samples were collected and identified as *Ulva lactuca* from the Abu-Qir shore along the Alexandria coastline in Egypt, where the water temperature ranged between 28.5 and 29.5 °C (September 2022) (Figure 1). Marine fungi were isolated by washing algae samples with sterile seawater, followed by homogenizing the thallus with 20 mL of seawater. A 1.0 mL aliquot of each homogenate was inoculated on three seawater agar media types (i.e., Rose Bengal chloramphenicol (RB), potato dextrose (PD), and Sabouraud Dextrose (SD)), with the pH adjusted to 5.0 ± 0.1 [12]. The plates were incubated at 30 ± 0.2 °C for 6 days, then the resulting pure fungal isolates were stored on (SD) at 4.0 °C for EPS screening [28].

### 2.2. Screening for EPS Production

Six fungal isolates (AG1, AG2, AG4, AG5, AG6, and AG7) were obtained from algae samples S1, S2, S4, S5, S6, and S7, respectively, and screened for their potential to produce EPS (Figure 1). These isolates were cultivated on a modified seawater SD broth medium containing 10.0 g/L of peptone and 20.0 g/L of dextrose (HiMedia Co., Mumbai, India), at 30 ± 0.2 °C and pH 5.0 ± 0.1 for 6 days in a static incubator. After the incubation, the culture filtrate was separated from mycelium by filter paper. The mycelium was dried overnight at 50 °C, and its weight was recorded. Proteins were precipitated using (10% *w*/*v*) trichloroacetic acid (TCA) for 30 min, and the resulting colloids were removed by centrifugation at 4500 rpm for 20 min. The supernatant was mixed with absolute ethanol in a 3:1 ratio (*v*/*v*) and stored overnight at 4.0 °C. The precipitated EPS was collected by centrifugation and dried for 24 h at 35 °C. The crude EPS was determined as the mean of three trials [29].

### 2.3. Genetic Identification of Fungal Isolate

The most potent isolate with the highest EPS yield (AG7) was cultivated on SD agar medium for 6 days. It was then identified based on macroscopic characteristics (colony color, texture, and mycelial habit, including whether the hyphae were aerial or growing within the medium) and microscopic features (conidiophores, vesicles, and conidia) through examination under a light microscope at 400×, following previously described methods [30]. Taxonomic guidelines from Barnett and Hunter (1986) were followed for accurate classification [31]. DNA extraction from the isolate was performed, followed by PCR amplification of the ITS regions of rDNA using two primers: ITS1 (**5′**TCCGTAGGTGAACCTGCGG**3′**) and ITS4 (**5′**TCCTCCGCTTATTGATATGC**3′**) [32]. The PCR products were purified with the AccuPrep PCR DNA Purification Kit, and subsequently sequenced by Macrogen Incorporation (Seoul, Republic of Korea). The sequences were deposited in GenBank under accession number of (OR552110.1) and analyzed using BLASTN for similarity at http://www.ncbi.nlm.nih.gov/BLAST (accessed on 19 September 2023). A Neighbor-joining phylogenetic tree was constructed using the MEGAX_10.1.6 program software [28,30]. High-quality reagents and chemicals were provided by AccuPrep PCR DNA Purification Kit (K-3034-1, Bioneer Corporation, Daejeon, Republic of Korea), DNA STAR SeqMan (DNA STAR Incorporation, Madison, WI, USA), and Sigma-Aldrich (St. Louis, MO, USA).

### 2.4. Purification and Structural Analysis of EPS

About 3.85 g of the crude fungal EPS-AG7 underwent several purification steps: first, deproteinization was performed using 10% (*w*/*v*) TCA overnight at 4 °C, resulting in the removal of precipitated proteins. The remaining soluble crude EPS-AG7 was then precipitated with cold ethanol, yielding 3.0 g of dried weight [33,34]. Subsequently, impurities and low molecular weight compounds were removed using a dialysis bag (cut-off 3500 Da) [35], which resulted in 1.18 g of semi-pure EPS-AG7 after lyophilization. The EPS was further fractionated on a sephadex-50 column using ultrapure water at a flow rate of 0.2 mL/min. All collected tubes were checked for carbohydrate using the phenol–sulfuric acid method [36]. UV absorbances measurements of the eluted fractions indicated a single Gaussian distribution curve. The fractions were collected, lyophilized, and yielded 0.52 g of pure EPS-AG7, which was stored in a plastic bag at 4.0 °C for structure identification and analysis.

#### 2.4.1. Carbohydrate and Sulfate Contents

The carbohydrate content (%) of EPS-AG7 was determined using the phenol–sulfuric acid method [36]. Briefly, 150 µL of a 5.0 mg/mL EPS-AG7 solution was mixed with 150 µL of 5.0% (*w*/*v*) phenol solution, followed by the addition of 750 µL of concentrated sulfuric acid. The mixture was cooled at room temperature before measuring absorbance at 490 nm using a UV-Visible spectrophotometer (Jasco V-630 BIO, Yokohama, Japan). Carbohydrate content (%) was calculated using a standard glucose curve [37]. The total sulfate content of EPS-AG7 was determined by the turbidimetric method [38]. Initially, 6.0 mg of purified EPS-AG7 was hydrolyzed with 2.0 mol/L hydrochloric acid (HCl), then diluted to 100 mL with distilled water (DW) and transferred to a 250 mL measuring flask. A volume of 5.0 mL of conditioning reagent, prepared by mixing glycerol (5.0 mL), concentrated HCl (3.0 mL), ethyl alcohol (10 mL, 95%), and sodium chloride (7.5 g), then completed to 100 mL with DW, was added. After stirring for 1.0 min, a few milligrams of BaCl_2_ fine powder were added, followed additional stirring for another 1.0 min and immediate absorbance measurement at 420 nm using a UV-Visible spectrophotometer. The total sulfate content was quantified against a standard curve generated using various sodium sulfate (Na_2_SO_4_) concentrations with BaCl_2_ [39].

#### 2.4.2. Structural Functionality

The chemical functional groups of the EPS-AG7 molecular structure were analyzed using FTIR spectrometer (Jasco FT/IR-4600 plus, Tokyo, Japan) employing the KBr pellet method [40,41]. About 1.0 mg of purified EPS-AG7 was mixed with KBr in a ratio of 1:100 (*w*/*w*) and ground to produce a homogeneous fine powder. This mixture was then pressed into a transparent disc of 1.0 mm thickness. The disc was scanned over 128 scans within the frequency range of ύ = 4000–400 cm^−1^.

#### 2.4.3. Molecular Structure Identification

The proton (^1^H) and carbon (^13^C) NMR experiments were performed using JEOL JNM-ECA600NMR (JEOL Ltd., Tokyo, Japan). The EPS-AG7 was dissolved in deuterium oxide (D_2_O, 99.96%) at a concentration of 40 mg/L. The solution was then lyophilized and redissolved in D_2_O for three cycles. The final EPS-AG7 sample in D_2_O was subjected to 1D and 2D NMR analysis at 25 °C to elucidate its molecular structure.

#### 2.4.4. Monosaccharides Composition

Identification and quantification of monosaccharides in EPS-AG7 were performed using the PMP-labeling method [42,43,44]. Briefly, 100 µL of a 5.0 mg/mL EPS-AG7 was hydrolyzed with 100 µL of 4.0 mol/L trifluoroacetic acid at 100 °C for 4 h. The hydrolysates underwent derivatization by adding 200 µL of ammonia solution and 200 µL of 1-phenyl-3-methyl-5-pyrazolone (PMP, 0.2 mol/L in methanol) at 70 °C for 1.0 h. The labelled sugar solution was then neutralized with 0.2 mmol/L hydrochloric acid to pH 7.0, while any unreacted PMP was extracted with chloroform (400 µL × 3 times). The aqueous phase was then lyophilized, re-dissolved in 250 µL DW, and filtered through a 0.2 µm syringe filter prior to analysis. For HPLC analysis, 20 µL of the derivatized sample was injected into HPLC system (UFLC Shimadzu, Japan) equipped with Kinetex^®^ 5 μm EVO C18 (Phenomenex, Inc., Torrance, CA, USA) (150 mm × 4.6 mm) maintained at 35 °C, with a UV detector set at λ = 254 nm. Separation of the PMP-derivatized sugars was achieved using an isocratic mobile phase consisting of phosphate buffer (0.1 mol/L; pH 6.7) and acetonitrile (83:17, *v*/*v*) at a flow rate of 0.7 mL/min. Monosaccharide standards (D-glucose, D-galactose, D-mannose, L-rhamnose, L-xylose, D-lyxose, L-ribose, D-arabinose, D-galacturonic acid, D-glucuronic acid, and L-fucose) underwent the same derivatization and extraction steps, while acid hydrolysis was not required [44]. Qualitative analysis of the sugar types in EPS-AG7 was established by comparing retention times with the corresponding standards, while quantification was based on peak areas and response factors for each standard.

#### 2.4.5. Methylation and Glycosidic Linkages Analysis

The determination of glycosidic linkages in EPS-AG7 was performed using the methylation method [45]. Approximately 6.0 mg of EPS-AG7 was mixed with NaOH (20 mg) and 1.0 mL of dimethyl sulfoxide (DMSO, Sigma-Aldrich, St. Louis, MI, USA) in a dry flask, followed by sonication for 1.0 h. Iodomethane (200 µL) was added, and the mixture was sonicated again for 1.0 h. After cooling in the dark, 1.0 mL of DW was added, and the methylated EPS was extracted three times with chloroform (1.0 mL each). The combined organic layers were washed trice with DW (1.0 mL each) and dried over anhydrous sodium sulphate. After drying under a stream of nitrogen, the methylated EPS residue was hydrolyzed with 2.0 mol/L TFA at 100 °C for 6.0 h. Following evaporation of TFA, the hydrolysates were reduced by adding sodium borohydride (NaBH_4_, 20 mg) for 3 h. The reaction was quenched and neutralized by adding acetic acid (0.5 mL, 25%), and the sample was evaporated overnight. The residue was dissolved in acetic anhydride (0.75 mL) and heated at 100 °C for 1.0 h. The acetylated-O-methylated sugar residues were extracted three times with chloroform (1.0 mL each). The collected organic phase was washed with DW, dried over sodium sulphate anhydrous, then analyzed using GC-MS (JMS-T100GCV AccuTOF GCv 4G, JEOL Ltd., Tokyo, Japan). The HP-5MS column (30 m × 0.25 mm ID × 0.25 μm) was used with a temperature program, as reported previously [46]. Masses spectra were acquired in electron ionization (EI) mode at 70 eV, and monomers’ identification was achieved by comparing fragmentation patterns (m/z) with NIST Library data.

#### 2.4.6. Homogeneity and Average Molecular Weight $(\bar{Mw)}$

The $(\bar{Mw)}$ and homogeneity of EPS-AG7 were estimated using an UFLC system (Shimadzu Corporation, Kyoto, Japan) equipped with a TSK-gel G4000SW column (7.5 mm × 300 mm) maintained at 30 °C, coupled with RI detector (RID-20A BLK, Shimadzu Corporation, Kyoto, Japan). A volume of 20 μL of EPS-AG7 (4.0 mg/mL) was injected and eluted using a Na_2_SO_4_ solution (50 mmol/L) at a flow rate of 1.0 mL/min. Determination of the $(\bar{Mw)}$ was based on calibration curve with dextran standards of known Mw (5, 10, 25, 50, 150, 270, and 410 kDa) [47].

#### 2.4.7. Conformation Analysis of EPS

##### Congo Red (CR) Test

The helical arrangement of some polysaccharides can be investigated by observing a bathochromic effect in the maximum absorption of CR when mixed with a polysaccharide in an alkaline medium [48,49]. A stock EPS-CR mixture was prepared by mixing 2.0 mL of EPS (2.0 mg/mL) with 2.0 mL of CR (182 μmol/L). Subsequently, 200 µL of this stock mixture was transferred into various tubes, and 50 µL of different concentrations of NaOH were added to adjust the final NaOH concentration in the range from 0 to 1.0 mol/L. Control samples containing only CR with varying NaOH concentrations were also prepared for comparison. All samples were monitored using a UV-Vis spectrophotometer in the wavelength range of 700 to 400 nm. The investigation into the helical arrangement was conducted by plotting the $(\lambda_{max};nm)\;$of each EPS-CR sample against [NaOH,mol/L] while comparing with the CR controls.

##### Circular Dichroism (CD) Analysis

The structural changes in EPS-AG7 induced by various factors were investigated using a vacuum-ultraviolet circular dichroism (VUV-CD) spectropolarimeter installed at BL-12, Hiroshima Research Institute for Synchrotron Radiation Science (HiSOR), Japan. Firstly, EPS-AG7 solutions were prepared in both Tris-HCl buffer (pH 7.6) and ultrapure water. Concentration-dependent experiments were conducted using EPS concentrations in the range of 0.5%–6.0% *w*/*v* in DW. For temperature-dependent studies, EPS-AG7 at a concentration of 6.0% *w*/*v* was analyzed across a temperature range of 20 °C to 65 °C using a temperature control unit and waiting for 1.0 min before monitoring [50]. The impact of ionic strength on EPS-AG7 structuration at a concentration of 2.5% (*w*/*v*) was explored using high concentration of sodium fluoride (NaF, 50 mmol/L) [51,52,53]. Additionally, interactions between EPS-AG7 and lipids were investigated using three types of lipid molecules: 1,2-dioleoyl-sn-glycero-3-phosphoethanolamine (DOPE), 1,2-dioleoyl-*sn*-glycero-3-phosphocholine (DOPC), and 1,2-dioleoyl-*sn*-glycero-3-phospho-L-serine (sodium salt) (DOPS). The liposomes, with a diameter of 100 nm, were prepared by the extrusion method [54,55]. Briefly, each lipid was suspended in Tris-HCl buffer (10 mmol/L, pH 7.6), vortexed and subjected to freezing by liquid N_2_ followed by heating at 65 °C for five cycles to ensure exceeding the phase-transition temperature ($T_{m}$) of the lipids ($T_{DOPC}=-17\;{}^{\circ}C,T_{DOPE}=-16\;{}^{\circ}C,{}^{\circ}and{}^{\circ}T_{DOPS}=-11\;{}^{\circ}C$). After that, the suspension was extruded 25 times using a Mini-Extruder (Avanti Polar Lipids, Inc., Alabaster, AL, USA) by the forward-back syringe injection of the suspension through a 100 nm polycarbonate membrane (Whatman, Clifton, NJ, USA). EPS-liposome samples in Tris-HCl buffer were prepared with varying lipid/EPS weight ratios (0, 5, 10, 25, 50, 100:1), incubated at 25 °C for at least 12 h before analysis, with the final EPS-AG7 concentration maintained at 2.5% (*w*/*v*).

Synchrotron radiation circular dichroism (SRCD) measurements were conducted using 20 µL of sample solution, which was sandwiched between two CaF_2_ cell windows. The SRCD spectra were acquired with a VUV-CD spectropolarimeter at BL-12, HiSOR (Higashi-Hiroshima, Japan) [56,57]. The spectra were recorded over six scans with a 0.67 nm step resolution, a path length of 50 µm, and a response time of 4 s, while the scanning speed was set at 20 nm/min, in the range of 170–250 nm (far-UV to VUV). The SRCD spectra were then background-subtracted to identify the structural changes associated with the inducing parameter [58,59].

### 2.5. Potential Activities of EPS-AG7

#### 2.5.1. Anticoagulant Activity

The prothrombin time (PT) and activated partial thromboplastin time (APTT) activities of EPS-AG7 were evaluated using (PT) and (APTT) assay kits (Biomed Diagnostics, Germany). The EPS-AG7 was prepared in DW at 6.0 mg/mL. Plasma was collected from whole blood samples using tubes containing sodium citrate (0.109 mol/L) as an anticoagulant, with a blood-to-anticoagulant ratio of 9:1. Plasma was separated by centrifuging at 4500 rpm for 15 min. For the PT activity, 100 µL of citrated plasma was mixed with 100 µL of EPS and incubated at 37 °C for 1.0 min; DW and heparin (1.0 IU/mL, Amoun Pharmaceutical Co., Al Obour City, Egypt) served as negative and positive controls, respectively. After adding 400 µL of the PT reagent and incubating at 37 °C for 10 s, clot formation time was recorded. For APTT, a mixture of 50 µL of citrated plasma and 50 µL of EPS-AG7 was incubated at 37 °C for one minute; DW and heparin (1.0 IU/mL) were used as controls. Following the addition of 50 µL of the APTT reagent and 1.0 min of incubation at 37 °C, the clotting time was measured after adding 50 µL of CaCl_2_ solution (50 μmol/L) [39].

#### 2.5.2. Antioxidant Activity

The antioxidant behavior of EPS-AG7 was evaluated using two methods: the 1,1-diphenyl-2-picryl hydrazyl radical (DPPH) scavenging method and the 2,2’-azino-bis(3-ethylbenzothiazoline-6-sulfonic acid)-radical (ABTS^+^) assay. For the DPPH, various concentrations of EPS-AG7 (10.0 to 0.50 mg/mL) were prepared in DW. Then, 0.2 mL of each concentration was mixed with DPPH solution (0.2 mL, 0.2 mmol/L in methanol), vortexed, and incubated in the dark for 15 min, followed by absorbance measurement at 517 nm using a UV-Visible spectrophotometer against a corresponding blank [60]. In the ABTS^+^ assay, a stock of ABTS^+^ solution was prepared by reacting ABTS^+^ (7.0 mmol/L) with ammonium peroxodisulfate (2.45 mmol/L) in dark for 12–16 h, then the formed cation radical (ABTS^+^) solution was diluted with ethanol to an absorbance of 0.70 ± 0.02 at 734 nm. Similarly, 0.2 mL of each EPS-AG7 concentration was mixed with ABTS^+^ solution (0.2 mL), incubated at room temperature for 6.0 min, and measured at 734 nm against a blank [61]. Ascorbic acid at concentrations (2.0 to 0.01 mmol/L) served as a positive control. The percentage inhibition of both radicals was calculated using the formula: $Scavenging\;\%=\frac{A_{0}-(A_{s}-A_{b})}{A_{0}}\times 100$, where $A_{0}$, $A_{S}$, and $A_{b}$ represent the absorbances of the radical solution, EPS sample mixed with the radical solution, and blank samples, respectively. The EC50 values of EPS-AG7, indicating the concentration required to inhibit 50% of the radicals, were obtained using a dose–response experiment.

## 3. Results

### 3.1. Screening of EPS Production by Marine Fungal Isolates

**Figure 2:**
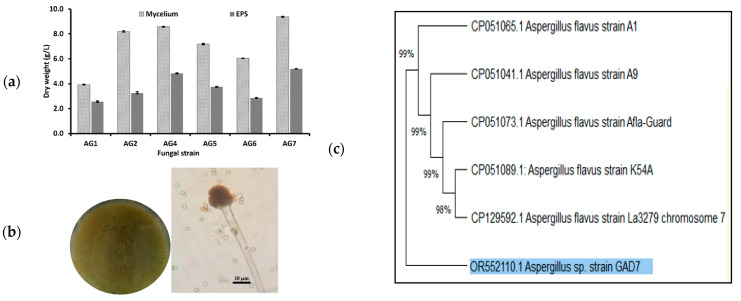
(**a**) *Mycelium* dry weights and EPS yields of fungal isolates, (**b**) macrographs showing macroscopic features on SD agar (**b**-**left**) and microscopy details of *Aspergillus* sp. strain GAD7 (**b-right**), and (**c**) phylogenetic tree of *Aspergillus* sp. strain GAD7.

Six EPS-producing fungal strains (AG1, AG2, AG4, AG5, AG6, and AG7) were isolated from marine algae samples S1, S2, S4, S5, S6, and S7, respectively, and screened for EPS production in SD media. All isolates produced EPS with yields ranging from 2.86 to 5.19 g/L and mycelium dry weights between 3.93 and 9.38 g/L. The AG7 isolate exhibited the highest EPS production (5.19 ± 0.017 g/L) and mycelium dry weight (9.38 ± 0.043 g/L), making it the most potent EPS producer and the subject for further study (Figure 2a).

### 3.2. Identification of the Marine Fungal AG7 Isolate

The AG7 isolate was identified morphologically and by molecular genetic as *Aspergillus* sp. strain GAD7 (Figure 2b). According to macroscopic characteristics, the strain GAD7 was exhibited as a radially spreading yellowish-green colony from the site of inoculation. The colony’s center slightly elevated as mycelia piled and displayed roughness. Four days later, sporulation began to emerge from the colony’s center and spread outward, covering the entire surface. The conidia were green to yellow, and the sporulating mycelia were surrounded by a white border. The microscopic characteristics of *Aspergillus* sp. strain GAD7 demonstrated that the phialides radiate outward from the vesicle on all sides. The walls of the conidia were thin and rough. The conidiophores were unbranched, thick-walled, and rough in texture (Figure 2b). The ITS sequence of the AG7 isolate was registered in GenBank under accession number (OR552110.1). The sequence showed ~97.40% similarity with *Aspergillus flavus* strain La3279 chromosome 7, represented by the phylogenetic tree relationships (Figure 2c).

### 3.3. Spectroscopic Studies of the EPS-AG7

#### 3.3.1. Carbohydrate and Sulfate Contents

The UV-Vis analysis showed a high carbohydrate content of ~87.5% as glucose following the phenol–sulfuric acid method [36]. Additionally, the EPS-AG7 sample was found to have approximately 24% sulfate content, determined using the turbidimetric method [38].

#### 3.3.2. Structural Functionalities

**Figure 3:**
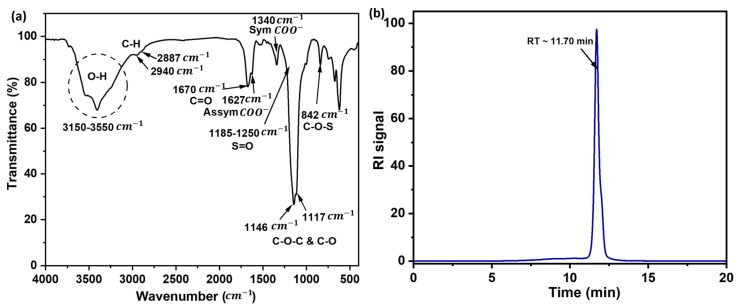
(**a**) FTIR spectrum, and (**b**) SEC chromatogram of the EPS-AG7 produced by *Aspergillus* sp. GAD7.

FTIR spectroscopy was applied to identify the structural functionalities of EPS-AG7. The IR spectrum of EPS-AG7 exhibited characteristic polysaccharide features in the “anomeric region” (950–750 ${cm}^{-1}$) and the “sugar region” (1200–950 ${cm}^{-1}$) [62]. It showed the stretching and bending vibrational bands related to O-H, C-H, C=O, C-O, and C-O-C groups [63,64] (Figure 3a). The spectrum also displayed multiple absorption bands at (ύ = 3150–3550 ${cm}^{-1}$), indicating hydroxy (-OH) groups of alcoholic and carboxylic nature, supported by two bands at ύ = 1340 and 1627 ${cm}^{-1}$ related to the symmetric and asymmetric vibrations of the carboxylate (${COO}^{-}$) group, respectively [65]. Weak absorption bands at ύ = 2887 and 2940 ${cm}^{-1}$ denoted the stretching vibration of the (C-H) in methyl groups, while a moderately intense peak at ύ = 1670 ${cm}^{-1}$ was characteristic of the carbonyl (C=O) group. The carbohydrate characteristic signature was evident by the sugar region (ύ = 1000–1200 ${cm}^{-1}$), with intense signals at ύ = 1117 and 1146 ${cm}^{-1}$ belonging to the stretching vibrations of the C-O and C-O-C groups in polysaccharides [62]. The signal around 842 ${cm}^{-1}$ suggested the presence of the C-O-S group, potentially associated with an overlapping signal at ύ = 1185–1250 ${cm}^{-1}$ resulting from the stretching vibration of the S=O in sulfate (${-OSO}_{3}^{-}$) group [66].

#### 3.3.3. Homogeneity and Average Molecular Weight of the EPS-AG7

The SEC technique was used to estimate the homogeneity and average molecular weights of the purified EPS [47]. The EPS-AG7 exhibited a single Gaussian distribution curve, indicating a single molecular weight distribution (Figure 3b). Using dextran standards, the average ($\bar{Mw}$) of the EPS-AG7 was approximately 7.34 × 10^3^ Da, calculated using the equation Log Mw=−0.3352 x+4.7877, where x is the time (min) (Figure S1; Supporting Information).

#### 3.3.4. Molecular Structure Identification of the EPS-AG7

The ^1^H NMR spectrum of EPS-AG7 revealed a complex EPS structure, with intense signals in the upfield ring protons region (δ = 3.12–4.35 ppm) and several signals in the downfield anomeric region (δ = 4.40–5.50 ppm) (Figure S2a; Supporting Information). Despite overlapping proton signals that impeded precise assignments, a signal at δ = 1.98 ppm was assigned to protons of acetyl groups in the polysaccharide, suggesting that some sugars are in an O-acetylated form [67]. The existence of multiple signals in the chemical shift region at δ = 5.0–5.50 ppm, compared to δ = 4.40–4.80 ppm, proposed that most monosaccharide moieties are connected through α-glycosidic than the β-type, respectively [68,69]. The ^13^C spectrum exhibited a high noise-to-signal (N/S) ratio, providing limited information on the carbon environments (Figure S2b; Supporting Information), indicating structural complexity and suggesting that EPS-AG7 exists in a stable folded conformation, as detected by SRCD measurement. No correlations were observed in 2D NMR experiments (i.e., COSY, HSQC, HMBC, and TOCSY), which hindered the prediction of the monosaccharides’ sequence and the molecular structure of EPS-AG7, (Figure S2c–f; Supporting Information).

#### 3.3.5. Monosaccharides Composition of the EPS-AG7

The HPLC analysis, using monosaccharide standards, identified various monosaccharides in EPS-AG7, revealing a heterogeneous composition. The molar percentage ratios were 42.14% mannose, 24.59% glucose, 11.96% arabinose, 8.27% galacturonic acid, 6.96% galactose, and 6.35% lyxose. Additionally, ribose was suggested to be present, as indicated by a very low intensity peak in the chromatogram, though its concentration was likely below the limit of detection (Table S1 and Figure S3; Supporting Information).

#### 3.3.6. Methylation and Glycosidic Analysis

The analysis identified several monosaccharides in EPS-AG7, including glucose, mannose, galactose, ribose, and lyxose, which were identified by comparing their mass spectra with the data from the NIST library (Table S2 and Figure S4; Supporting Information). Furthermore, it was observed that sugar units in EPS-AG7 are linked together through both α- and β-glycosidic bonds, indicating structural diversity in the linkage patterns of the polysaccharide.

#### 3.3.7. Conformational Analysis of the EPS-AG7

The UV-Vis and intrinsic SRCD experiments were carried out to study the conformation and structuration of the EPS-AG7 produced by *Aspergillus* sp. GAD7.

##### Congo Red (CR) Assay

**Figure 4:**
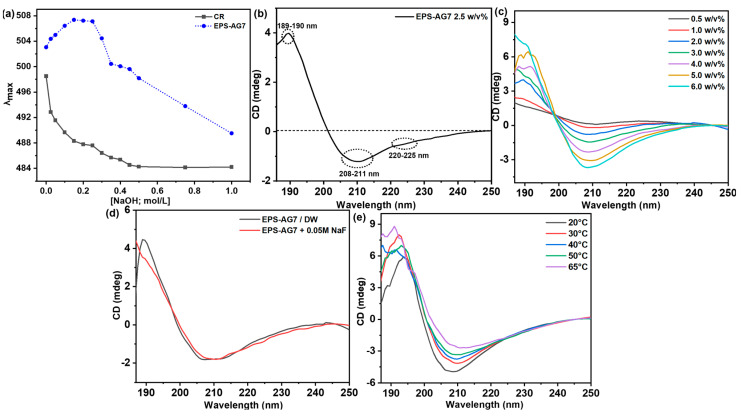
(**a**) Plot of $\lambda_{max}$ of CR-EPS against [NaOH] using UV-Vis, (**b**) VUV-CD spectrum of EPS-AG7 by *Aspergillus* sp. GAD7 (2.5 *w*/*v*% in DW, 25 °C); (**c**) VUV-CD concentration dependent of EPS-AG7 (0.5–6.0 *w*/*v*% in DW); (**d**) VUV-CD spectra in absence and presence of NaF (50 mmol/L); and (**e**) VUV-CD temperature-dependent spectra of EPS-AG7 (6.0 *w*/*v*%, 20 °C–65 °C in DW).

The $\lambda_{max}$ of the CR dye was monitored in the presence and absence of EPS-AG7, and the data were plotted against NaOH concentrations, as shown in Figure 4a. Initially, the $\lambda_{max}$ of CR was shifted to longer wavelengths as NaOH concentrations increased up to 0.25 mol/L. This shift suggested that EPS-AG7 might adopt a triple helix arrangement at NaOH concentrations below 0.25 mol/L. Subsequently, as NaOH concentration increased within the range of 0.25 to 0.50 mol/L, there was a gradual decrement in the $\lambda_{max}$ of CR. This observation indicated a gradual denaturation of the helix-like structure of EPS-AG7 under these conditions. Further increases in NaOH concentration beyond 0.5 mol/L led to the complete denaturation of the helical structure of EPS-AG7, characterized by the minimum $\lambda_{max}$ values [18,20,70]. To gain more detailed insights into the helical arrangement of EPS-AG7, SRCD measurements were conducted using a CD spectropolarimeter.

##### Synchrotron Radiation Circular Dichroism (SRCD) Analysis

The CD spectrum of EPS-AG7 in DW showed broad negative Cotton effects with one relatively small and one high intensity bands in the ranges of 220–225 and 208–211 nm with extrema at $\lambda_{ext}$ = 222 nm and $\lambda_{ext}$ = 209 nm, respectively (Figure 4b). Additionally, a positive Cotton effect of relatively higher intensity was observed around 189–190 nm with an extremum at $\lambda_{ext}$ = 189.5 nm. This CD signature suggested that EPS-AG7 adopted a helical arrangement in DW [71], consistent with the findings from CR experiment.

**Figure 5:**
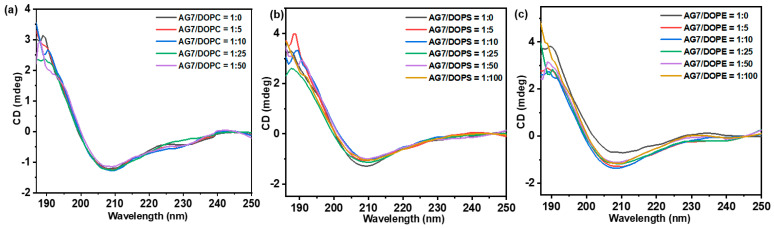
VUV-CD spectra of EPS-AG7/lipid at different weight ratios (*w*/*w*%): (**a**) EPS-AG7/DOPE; (**b**) EPS-AG7/DOPS; and (**c**) EPS-AG7/DOPC, in Tris-HCl buffer of pH 7.6 at 25 °C, and EPS-AG7 concentration of 2.5 *w*/*v*%.

This helical arrangement appeared stable under various experimental conditions. Concentration-dependent experiments (0.5–6.0 *w*/*v*%) in DW revealed increases in the negative and positive ellipticities of EPS-AG7 at $\lambda_{ext}$ = 209 nm and $\lambda_{ext}$ = 189.5 nm, respectively, without notable peak shifts (Figure 4c). The CD spectra of EPS-AG7 showed an identical structural arrangement in the absence and presence of a high NaF concentration (50 mmol/L), suggesting stability of the secondary structure of EPS-AG7 through intramolecular forces (Figure 4d). In contrast, temperature-dependent experiments revealed a slight decrease in the ellipticities of the Cotton effects when the temperature was raised 20 °C to 65 °C (Figure 4e). Regarding the influence of lipids (DOPE, DOPS, and DOPC) on the structuration of EPS-AG7 at different lipid/EPS weight ratios (i.e., 0, 5, 10, 25, 50, 100: 1.0 *w*/*w*%), Figure 5a–c showed that all lipids at the various ratios did not induce significant changes in the helical arrangement of EPS-AG7. The CD spectra of all samples closely resembled that of EPS-AG7 alone; however, DOPE might have caused a slight decrease in the intensity of the CD band at λ = 208–210 nm.

### 3.4. Structure–Activity Relationship

#### 3.4.1. Anticoagulant Activity of EPS-AG7

To evaluate the anticoagulant activity of EPS-AG7, PT and APTT assay kits were employed. The study assessed the efficacy of EPS across different genders and age groups, encompassing a total of 30 cases: 10 cases each for men, women, and children (Table 1).

In males, the average PT values increased from 14.29 s to 21.58 s, resulting in an increase in the international normalized ratio (INR) from 1.153 s to 2.14 s and a decrease in clotting factor concentration from 85% to 41.25%. Similarly, in females, the average PT values increased from 14.28 s to 21.44 s, with an increase in the INR from 1.151 s to 2.12 s and a decrease in the concentration from 85% to 41.75%. Among children, the average PT values increased from 13.68 s to 21.64 s, leading to an increase in the INR from 1.079 s to 2.14 s and a decrease in clotting factor concentration from 91.5% to 41.0%. The results collectively demonstrated that EPS-AG7 at a final concentration of 3.0 mg/mL doubled the blood coagulation times (APTT) from 29.9 s to 60.4 s in males, 29.2 s to 59.5 s in females, and 27 to 60.8 s in children (Table 1). In this study, the negative control (DW) used in PT and APTT analyses showed no significant difference compared to normal healthy individuals.

Moreover, the EPS produced from *Aspergillus* sp. strain GAD7 exhibited a more pronounced PT and APTT effect than the positive control heparin across all genders and age groups under the mentioned experimental conditions. These findings indicate that EPS from *Aspergillus* sp. strain GAD7 possesses an anticoagulant activity across different genders and ages.

#### 3.4.2. Antioxidant Activity of EPS-AG7

**Figure 6:**
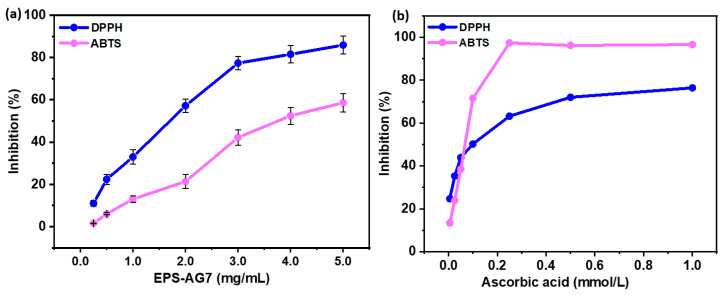
DPPH and ABTS^+^ free radicals scavenging activity (%) of (**a**) EPS-AG7 at a final concentration range of 0.25–5.0 mg/mL, versus (**b**) ascorbic acid at a final concentration range of 0.005–1.0 mmol/L (0.88–176.12 µg/mL).

The DPPH and ABTS^+^ methods are widely used to estimate the free radical scavenging potential of polysaccharides [72]. In the current study, EPS-AG7 demonstrated concentration-dependent scavenging activity across the concentration range of 0.25 to 5.0 mg/mL. At 5.0 mg/mL, EPS-AG7 exhibited 85.90% ± 4.26% scavenging against DPPH radicals, with an EC50 value of 1.40 ± 0.10 mg/mL (~0.191 mmol/L) (Figure 6). For ABTS^+^ radicals, EPS-AG7 showed a maximum scavenging of 58.64% at 5.0 mg/mL, with an EC50 of 3.80 mg/mL (~0.518 mmol/L). Comparatively, ascorbic acid, used as a positive control, exhibited 76.40% scavenging against DPPH radicals and 96.62% scavenging against ABTS^+^ radicals, with EC50 values of 0.081 mmol/L and 0.058 mmol/L, respectively (Figure 6b).

## 4. Discussion

Marine fungi represent a promising source of novel bioactive compounds due to their unique adaptations to environmental factors like temperatures, salinity, and nutrients, fostering distinctive secondary metabolic processes [73,74]. Compared to terrestrial fungi, which have been extensively studied for their medicinal and biotechnological applications, marine fungi remain comparatively underexplored [75]. Understanding the potential applications of bioactive compounds, such as EPS, depends on their physicochemical and structural characteristics, which are intricately linked with their complex and diverse structures. The structural diversity of EPS is influenced by factors such as monosaccharide composition, glycosidic linkages, molecular weight, conformation, etc. [1,3]. Previous studies have highlighted *Aspergillus* sp. capability to secrete macromolecules such as proteins, polysaccharides [76,77], and secondary metabolites like enzymes, vitamins, phenolics, etc. [78,79,80]. Therefore, the current study focused on optimizing production, and conducting detailed structure and conformation analyses of EPS synthesized by *Aspergillus* sp. strain GAD7.

Among all screened fungal strains, which revealed high potential for EPS production, the EPS-AG7 isolate achieved the highest production at 5.19 ± 0.017 g/L, accompanied by the highest mycelium dry weight of 9.38 ± 0.043 g/L. The AG7 isolate was identified morphologically and genetically as *Aspergillus* sp. strain GAD7. Amer et al. (2020) reported a similar EPS yield of 4.98 g/L from *Aspergillus terreus* SEI [10], while Costa et al. (2019) reported only 1.34 g/L from *A. terreus* under different fermentation conditions [22].

Structurally, EPS-AG7 was characterized by high carbohydrate content and a significant percentage of sulfate, indicating efficient extraction and purification methods and confirming its sulfated nature. FTIR spectroscopy identified EPS-AG7 as an acidic polysaccharide with sulfated backbone, evidenced by characteristic carboxylate and sulfate groups [65]. SEC analysis indicated EPS-AG7 as predominantly homogeneous polysaccharide, showing a single Gaussian distribution curve and suggesting uniform molecular weight distribution. The calculated ($\bar{Mw}$) of EPS-AG7 (~7.34 × 10^3^) was lower than other fungal EPS namely SEI-EPS (~5.0 × 10^4^ Da) and AT-EPS (~1.8 × 10^4^ Da), which were produced by *A. terreus* in two separate studies [12,22]. At the molecular level, the ^1^H NMR spectrum of the EPS-AG7 revealed a complex EPS structure with intense signals in both the upfield ring region and downfield anomeric area. The results suggested the presence of some sugar residues as O-acetylated form [67], and most of the monosaccharide moieties are connected through α-glycosidic [68,69]. The ^13^C spectrum showed a high noise-to-signal (N/S) ratio, indicating structural complexity and a stable folded conformation of EPS-AG7. The 2D NMR analysis of EPS-AG7 did not yield sufficient correlations, which hindered the determination of the sequence of monosaccharides. HPLC analysis indicated EPS-AG7 is a heterogenous polysaccharide composed of various monosaccharides at different molar ratios, with the presence of galacturonic acid, confirming its acidic nature as shown in FTIR results. The GC-MS data further characterized EPS-AG7 as a complex polysaccharide primarily composed of glucose, mannose, galactose, ribose, and lyxose as main sugars linked together through α- and β-glycosidic bonds. The NMR and GC-MS could not provide enough information to predict the molecular and sugar sequence of EPS-AG7 due to its structure complexity. The conformational analysis of EPS-AG7 was performed using UV-Vis and intrinsic SRCD experiments. CR assay indicated that EPS-AG7 forms a triple helix-like conformation, as evidenced by a shift for the $\lambda_{max}$ of CR to a longer wavelength in the UV-Vis spectra [48,49,81]. As NaOH concentrations increased to 0.25 mol/L, the $\lambda_{max}$ of CR shifted to longer wavelengths, suggesting that EPS-AG7 may adopt a stable triple helix arrangement at these concentrations [18,20,70]. However, at NaOH concentrations exceeding 0.25 mol/L, the hydrogen bonds that stabilize the EPS-CR complex and the triple helix conformation partially dissociated, resulting in the formation of double and single helical structures. SRCD measurements suggested that EPS-AG7 adopts a helical arrangement in DW [71], corroborating the CR experiment results. This helix-like arrangement remained stable under different experimental conditions, including different concentrations in DW and high NaF concentration, indicating that the conformation of EPS-AG7 is maintained through intramolecular forces, and is not disturbed by high concentrations of counter ions. On the other hand, high temperatures may affect the CD signature of EPS-AG7, as increasing the temperature can break some of the weak inter- and intramolecular hydrogen bonds that stabilize its conformation, potentially leading to structural changes. In other studies, xanthan polysaccharide exhibited disorder–order conformational changes induced by ionic strength and temperature influences [51,52,53]. Previous studies have examined the impact of lipid membrane bilayers on the conformation of biomolecules, particularly proteins and polypeptides, using CD techniques [82,83]; however, no such studies have been conducted on polysaccharides. In this study, varying lipid/EPS (*w*/*w*) ratios did not significantly alter the helical arrangement of EPS-AG7. All samples displayed nearly identical CD spectra to EPS-AG7; however, DOPE caused a slight decrease in the intensity of the CD band at λ = 208–210 nm, likely due to favorable interactions between the charged, small polar head group of DOPE and the acidic EPS-AG7, compared to the bulky head groups of DOPC and DOPS.

The structure of microbial EPS from diverse sources varies in monosaccharide composition, molar ratios, glycosidic linkages, and molecular weights, influenced by genetic makeup, nutritional media, carbon sources, and production conditions. These structural differences affect their potential applications in biotechnological and biomedical fields. In the current study, the structural analysis of the EPS produced by *Aspergillus* sp., indicated that EPS-AG7 is heterogeneous acidic polysaccharide with a relatively high sulfate content (24%). EPS-AG7 is primarily composed of mannose, with a considerable mol% of galacturonic acid and arabinose, and it adopts a triple helix-like conformation in both DW and Tris-HCl buffer. Subsequently, EPS-AG7 was tested for antioxidant and anticoagulant activities. Anticoagulant activity (PT and APTT assays) indicated that EPS produced from *Aspergillus* sp. strain GAD7 has anticoagulant activity across all genders and ages, demonstrating its effectiveness in vitro. Anticoagulant activities have been reported for fungal EPS; for instance, Han et al. (2005) produced an EPS (YCP) from *Keissleriella* sp. YS4108, which exhibited clear anticoagulant activity evaluated by APTT and PT tests [84]. Mendes et al. (2009) produced EPS called Botryosphaeran (EPS_FRU_) from the fungus *Botryosphaeria rhodina* MAMB-05, and reported significant in vitro anticoagulant activity for its sulfated analogue, while EPS_FRU_ itself did not exhibit any anticoagulation property [85]. Moreover, Amer et al. (2020) found that the marine *A. terreus* EPS (SEI-EPS) could increase APTT from 33 s to 56 s and 251 s at concentrations of 10 and 100 mg/mL, respectively [10]. Additionally, Martinichen-Herrero et al. (2005) evaluated the anticoagulant activity of β-glucan produced by the lichen *Parmotrema mantiqueirense* Hale, and its sulfated form (β-glucan-SO_4_), finding that only the sulfated analogue exhibited good anticoagulant activity [86]. Similarly, Brandi et al. (2011) noted that botryosphaeran (EPSGLC-RS) at concentrations of 30 and 40 µg/mL in plasma showed anticoagulant activity of ~6.8 and ~10 times greater than control samples, respectively [87]. In the current study, EPS-AG7 demonstrated anticoagulant activity without the need for additional sulfonation, highlighting its potential as a promising anticoagulant agent. Our results revealed that EPS-AG7′s anticoagulant activity surpasses that of heparin under the experimental conditions, suggesting a new and promising alternative for anticoagulant therapy. On the other hand, the antioxidant assays showed that EPS-AG7 exhibits significant antioxidant activity, with maximum scavenging activities of 85.90% and 58.64% in DPPH and ABTS^+^ at 5.0 mg/mL, and EC50 values of 1.40 mg/mL and 3.80 mg/mL, respectively. The EC50 values were higher than ascorbic acid in DPPH (0.191 mmol/L versus 0.081 mmol/L), and ABTS^+^ (0.518 mmol/L *versus* 0.058 mmol/L); however, EPS-AG7 still demonstrates a promising level of antioxidant potency when compared to other polysaccharides. For instance, EPS-AG7 demonstrates higher antioxidant activity than *Lysobacter soyae* CJ11T [88], *Bacillus thuringiensis* RSK CAS4 [89], *Bacillus cereus* SZ1 [90], *Bacillus amyloliquefaciens* GSBa-1 [91], *Enterococcus faecium* BDU7, and *Lentinus velutinus* EPS. Specifically, *B. cereus* SZ1 showed only 54.0% DPPH scavenging at 3.0 mg/mL [90], while *E. faecium* BDU7 displayed a maximum of 63.50% at 8.0 mg/mL [92], both significantly lower than EPS-AG7. Conversely, some polysaccharides showed higher or comparable antioxidant activities to EPS-AG7. *Brasenia schreberi* (BPL-2) nearly achieved 100% ABTS^+^ scavenging at 0.8 mg/mL with an IC50 of 0.239 mg/mL [93], outperforming EPS-AG7. Polysaccharide N1 from *Aspergillus versicolor* N2bc showed stronger DPPH scavenging, with a maximum of 89.79% at 8.0 mg/mL and an EC50 of 0.97 mg/mL [93]. Additionally, the EPS from *Lactobacillus plantarum* KX041 possessed 96.5% of ABTS^+^ scavenging at 0.6 mg/mL with an EC50 of 0.2 mg/mL [94]. The TFu-AATPE polysaccharide derived from *Lentinus edodes* demonstrated DPPH and ABTS^+^ scavenging activities of 90.67% and 81.92%, respectively, at 3.0 mg/mL [95]. In contrast, polysaccharides from *Dendrobium officinale* and *Weissella cibaria* SJ14 (EPS-1) displayed comparable antioxidant activities, with *D. officinale* showing a maximum DPPH scavenging of 85.42% at 4.0 mg/mL [96] and *W. cibaria* SJ14 achieving 82.0% DPPH scavenging at 5.0 mg/mL with an IC50 of 1.42 mg/mL [97]. Thus, EPS-AG7 shows promising antioxidant potency, particularly in the DPPH assay, suggesting its potential in applications requiring DPPH radical scavenging. However, its efficacy in ABTS^+^ assay is lower than some polysaccharides and ascorbic acid, indicating variability in antioxidant performance based on the specific assay, which is linked to differing scavenging mechanisms. The antioxidant activity of EPS-AG7, attributed to its low molecular mass (7.34 kDa) [98,99], underscores its potential as an effective antioxidant agent in specific applications.

## 5. Conclusions

The current research achieved significant EPS production (~5.19 ± 0.017 g/L (and detailed structural and conformational analysis of EPS produced by a marine-derived strain, which was identified as *Aspergillus* sp. GAD7, with an accession number of OR552110.1. The strain was grown in seawater SD broth medium at 30 ± 0.2 °C and pH 5.0 ± 0.1 for 6 days. Structural analysis using UV-Vis and FTIR demonstrated the high purity (87.5%) and sulfated nature (24%) of EPS-AG7, while SEC estimated its molecular weight ($\bar{Mw}$) to be ~7.34 × 10^3^ Da. HPLC and GC-MS analyses revealed a heterogeneous and acidic EPS consisting of various monosaccharides, with α-glycosidic linkages being more prevalent than β- linkages, as confirmed by the NMR. Additionally, UV-Vis technique through the (CR) experiment revealed that EPS-AG7 adopted triple helix-like conformation as ascertained by CD studies in water and Tris-HCl buffer, and this conformation was stable under different experimental conditions. EPS-AG7 demonstrated a significant antioxidant activity, particularly in the DPPH assay compared to the ABTS^+^ assay, and also emerged as a promising anticoagulant agent.

## Figures and Tables

**Table 1:** Anticoagulant activity of EPS-AG7 on different blood samples including both age and gender of the blood source.

Parameter/Case	Children	Male	Female
Number of cases	10	10	10
Age (Year)	2–10	30–60	25–55
PT of healthy individuals (Second, s) (Mean value)	13.68 ± 0.09	14.29 ± 0.1	14.28 ± 0.07
INR of NHI (Mean value; s)	1.07	1.15	1.15
Concentration (Mean value; %)	91.5	85	85
PT of NHI (s) after adding EPS (Mean value; s)	21.64 ± 0.05	21.58 ± 0.2	21.44 ± 0.08
INR of NHI after adding EPS (Mean value; s)	2.14	2.14	2.12
Concentration (Mean value; %)	41.0	41.25	41.75
PT of DW (s) (Negative control)	12.93 ± 0.04	13.71 ± 0.1	13.84 ± 0.07
PT of heparine (s) (Positive control)	18.08 ± 0.03	17.93 ± 0.03	17.90 ± 0.05
APTT of NHI (Mean value; s)	27.0 ± 0.4	29.9 ± 0.6	29.2 ± 0.2
APTT of NHI after adding EPS (Mean value; s)	60.8 ± 0.9	60.4 ± 0.4	59.5 ± 0.9
APTT of DW (s) (Negative control)	26.05 ± 0.12	27.2 ± 0.8	27.7 ± 0.3
APTT of heparine (s) (Positive control)	47.2 ± 0.6	48.6 ± 1.0	47.6 ± 0.9

PT: prothrombin time; INR: international normalized ratio; APTT: activated partial thromboplastin time; NHI: normal healthy individuals.

## Data Availability

The original contributions presented in the study are included in the article/Supplementary Materials; further inquiries can be directed to the corresponding authors.

## Supplementary Materials

The following supporting information can be downloaded at: https://www.mdpi.com/article/10.3390/jof10090659/s1, Figure S1: The Dextran calibration curve depicts elution time as a function of varying molecular weights (5, 10, 25, 50, 150, 270, and 410 kDa) using HPLC (2.0 mg/mL in Na_2_SO_4_ solution of 50 mmol/L, TSK-gel G4000SW column, RI detector, 1.0 mL/min, 30 °C); Figure S2: (a) ^1^H NMR, (b) ^13^C NMR, (c) COSY, (d) HSQC, (e) HMBC, and (f) TOCSY spectra of the EPS-AG7 produced by *Aspergillus* sp. strain GAD7 (40 mg/mL in D_2_O at 298K); Figure S3: HPLC chromatograms for monosaccharides of the EPS-AG7 produced by *Aspergillus* sp. strain GAD7 compared with monosaccharide standards. Peaks: 1: mannose; 2: lyxose; 3: ribose; 4+5: rhamnose+glucuronic acid; 6: galacturonic acid; 7: glucose; 8: galactose; 9: xylose; 10: arabinose; 11: fucose; Figure S4: GC chromatogram for monosaccharides of the EPS-AG7 produced by *Aspergillus* sp. strain GAD7; Table S1: Analytical linearity of HPLC-UV of different reducing sugars-PMP derivatives; Table S2: Methylation analysis data of EPS-AG7.
