# Supplementary material for: Potential Bioactivities, Chemical Composition, and Conformation Studies of Exopolysaccharide-Derived Aspergillus sp. Strain GAD7
Source: J Fungi (Basel). 2024 Sep 19;10(9):659. doi: 10.3390/jof10090659 (PMC11432975; doi:10.3390/jof10090659)
Supplement: Supplementary file 1 [file jof-10-00659-s001.zip › jof-3129276-supplementary.pdf]

Supporting Data

Potential bioactivities, chemical composition, and conformation studies of exopolysaccharide-derived *Aspergillus* sp. *s*train GAD7

Mohamed I.A. Ibrahim[1,2*], Hassan A.H. Ibrahim[2], Tatsuki Haga[1], Atsuhiko Ishida[3], Tatsuo Nehira[4], Koichi Matsuo[1,5,6*], Ahmed M. Gad[2*]

[1] Research Institute for Synchrotron Radiation Science, HiSOR, Hiroshima University, Higashi-Hiroshima, Hiroshima 739-0046, Japan.
[2] National Institute of Oceanography and Fisheries (NIOF), Cairo, Egypt.
[3] Laboratory of Molecular Brain Science, Graduate School of Integrated Sciences for Life, Hiroshima University, Higashi-Hiroshima, Hiroshima 739-8521, Japan.
[4] Graduate School of Integrated Sciences for Life, Hiroshima University, Higashi-Hiroshima, Hiroshima 739-8521, Japan.
[5] International Institute for Sustainability with Knotted Chiral Meta Matter (WPI-SKCM[2]), Hiroshima University, Higashi-Hiroshima, Hiroshima 739-8526, Japan.
[6] Research Institute for Semiconductor Engineering, Hiroshima University, Higashi-Hiroshima, Hiroshima 739-8527, Japan.

*** Correspondence:** ibra2020@hiroshima-u.ac.jp; ibrahimmohamed2030@gmail.com; m.ibrahim@niof.sci.eg; or pika@hiroshima-u.ac.jp; or a_gad7000@yahoo.com

Table S1. Analytical linearity of HPLC-UV of different reducing sugars-PMP derivatives.

**PMP-Sugar derivative**	**Linear regression** **y = ax + b**	**R^2**
Mannose	y = 50188x – 586555	0.998
Lyxose	y = 111148x – 1.0 E+06	0.9991
Ribose	y = 163674x – 2.0 E+06	0.9991
Galacturonic acid	y = 126445x – 2.0 E+06	0.9956
Glucose	y = 27646x – 384183	0.9872
Galactose	y = 111544x – 1.0 E+06	0.9897
Arabinose	y = 118012x – 2.0 E+06	0.9759

a: Co-efficient of the linear regression; b: intercepts of the linear regression; y means chromatographic response area; x represents the concentration (µg/mL).

Calibration curves included the linear equations and R^2 of sugars in a series of mixed monosaccharide concentrations determined in the current study:

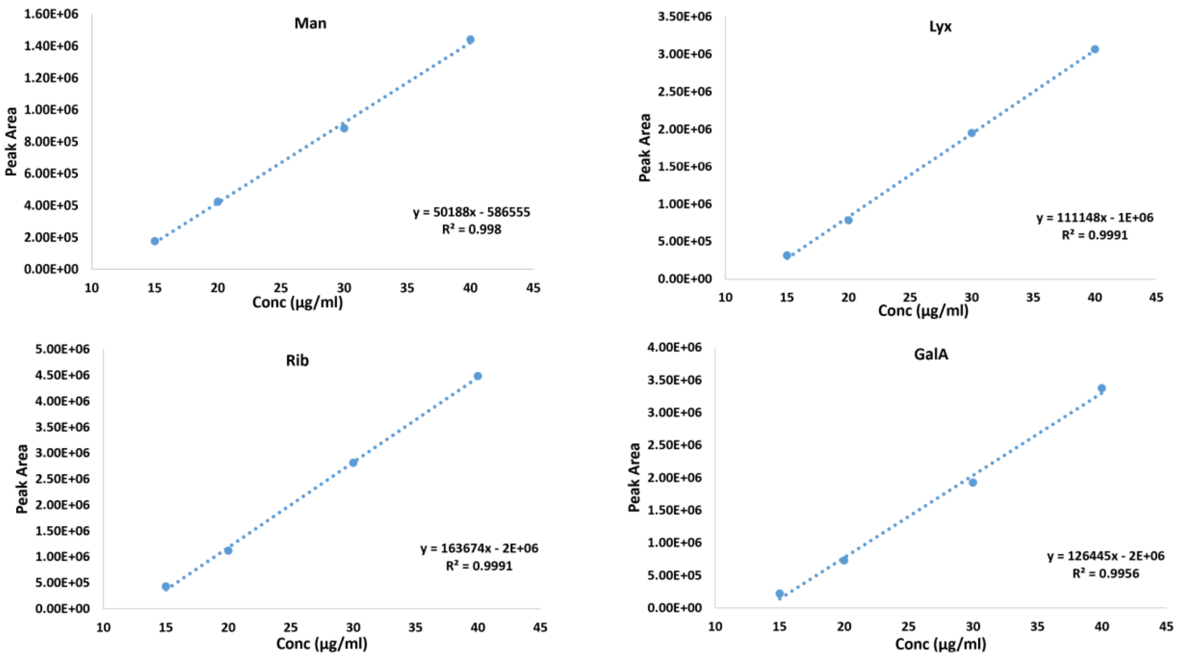

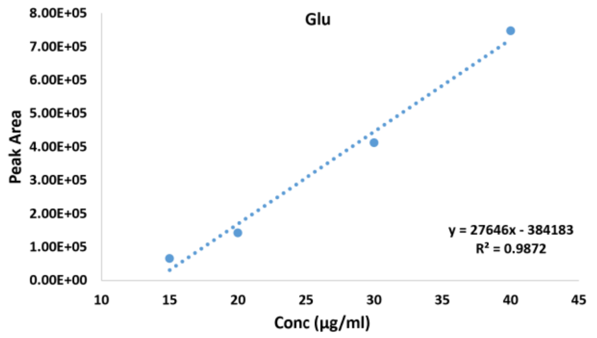

Glu
Peak Area
Conc (µg/ml)
y = 27646x - 384183
R² = 0.9872

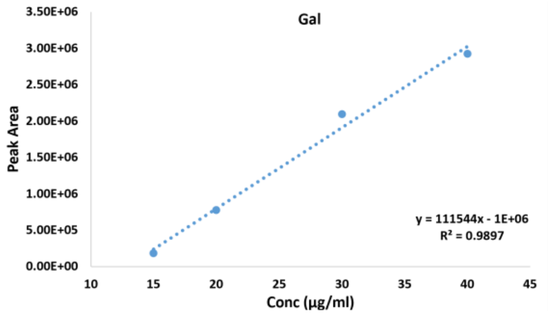

Gal
Peak Area
Conc (µg/ml)
y = 111544x - 1E+06
R² = 0.9897

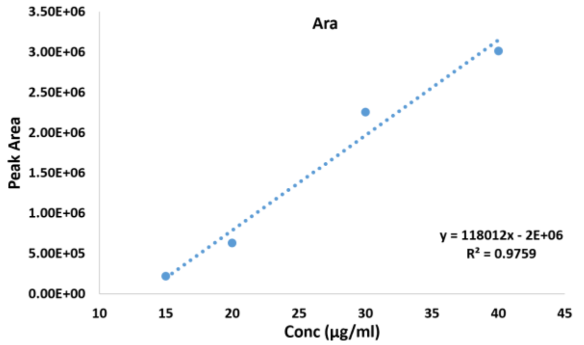

Ara
Peak Area
Conc (µg/ml)
y = 118012x - 2E+06
R² = 0.9759

Table S2. Methylation analysis data of EPS-AG7

Peak	RT (min)	Methylated sugars	Linkages type	Mass fragments (m/z)
1	13.02	2,3,4-Me3-Glu*p*	→6-α-D-Glu*p*-1→	43, 45, 59, 71, 74, 85, 88, 101, 129, 145
2	13.30	2,4,6-Me3-Man*p*	→3-β-D-Man*p*-1→	43, 45, 59, 71, 74, 87, 88, 101, 102, 129, 145, 159, 173
3	14.37	2,3,4,6-Me4-D-Glu*p*	D-Glu*p*-1→	43, 45, 72, 87, 101, 117, 129, 145, 161, 205
4	14.41	1,3,4,5-Me4-Man*p*	→6-β-D-Man*p*-2→	43, 45, 71, 87, 101, 117, 129, 145, 161, 205
5	14.53	1,3,4,5-Me4-Gal*p*	→6-α-D-Gal*p*-2→	43, 45, 71, 87, 101, 117, 129, 145, 161, 205
6	14.79	2,3,4-Me3-Gal*p*	→6-α-D-Gal*p*-1→	43, 45, 71, 87, 99, 101, 117, 129, 145, 161, 205
7	16.28	1,3,4-Me3-Rib*p*	D-Rib*p*-2→	43, 57, 71, 87, 101, 113, 117, 129, 135, 161, 189
8	16.59	1,2,4-Me3-Lyx*p*	D-Lyx*p*-3→	43, 57, 71, 87, 99, 101, 117, 129, 161, 189

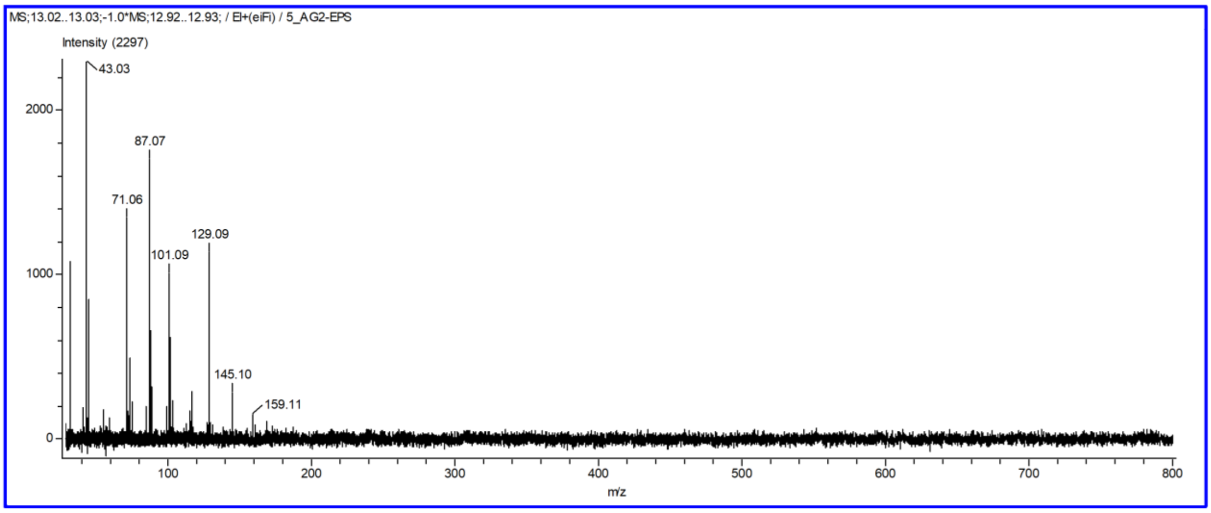

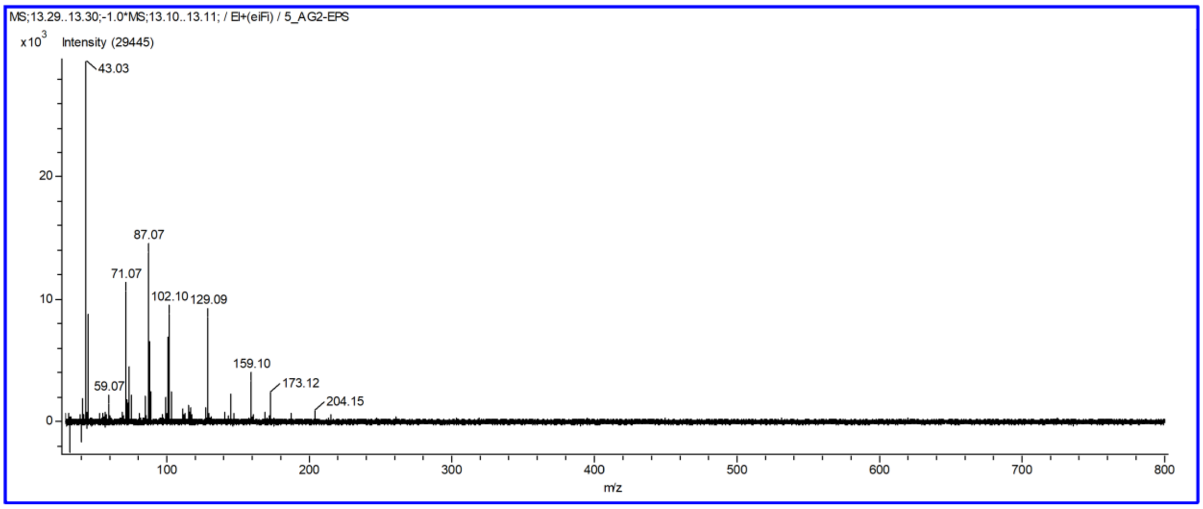

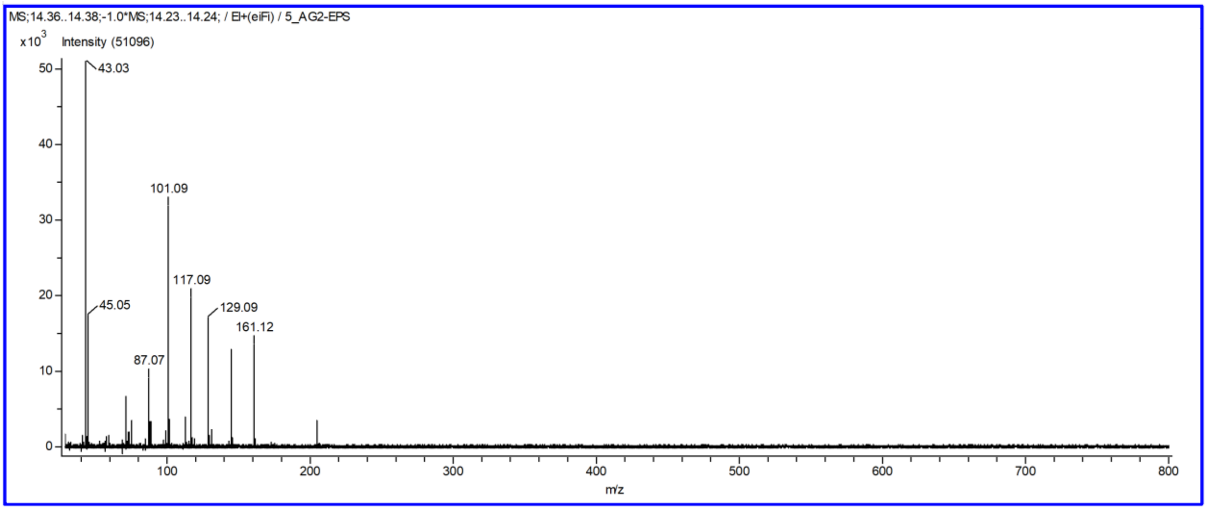
MS;14.36..14.38;-1.0*MS;14.23..14.24; / EI+(eiFi) / 5_AG2-EPS
x10^3 Intensity (51096)
43.03
101.09
117.09
45.05
129.09
161.12
87.07
m/z

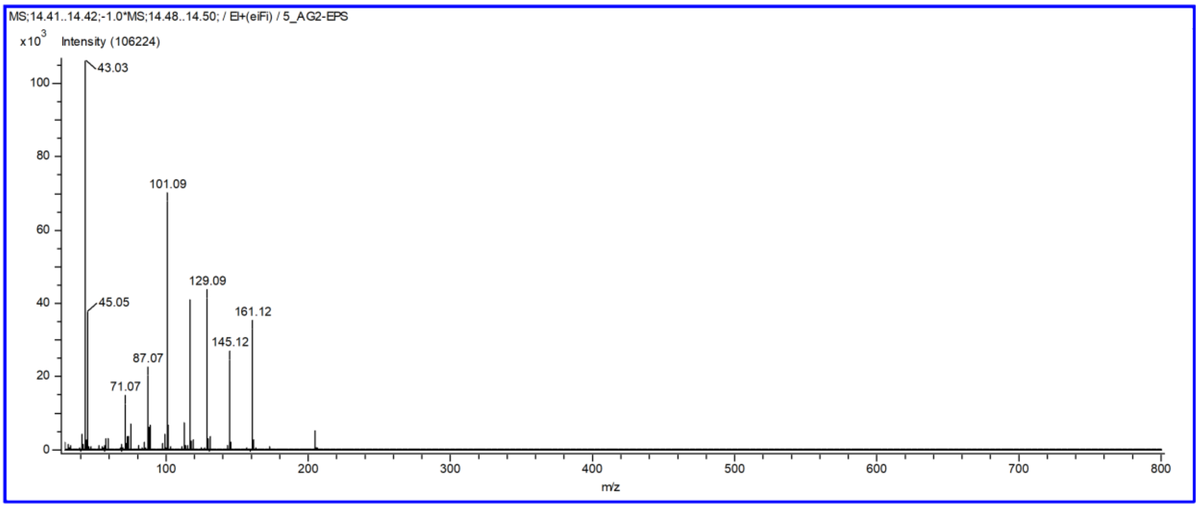
MS;14.41..14.42;-1.0*MS;14.48..14.50; / EI+(eiFi) / 5_AG2-EPS
x10^3 Intensity (106224)
43.03
101.09
129.09
45.05
161.12
145.12
87.07
71.07
m/z

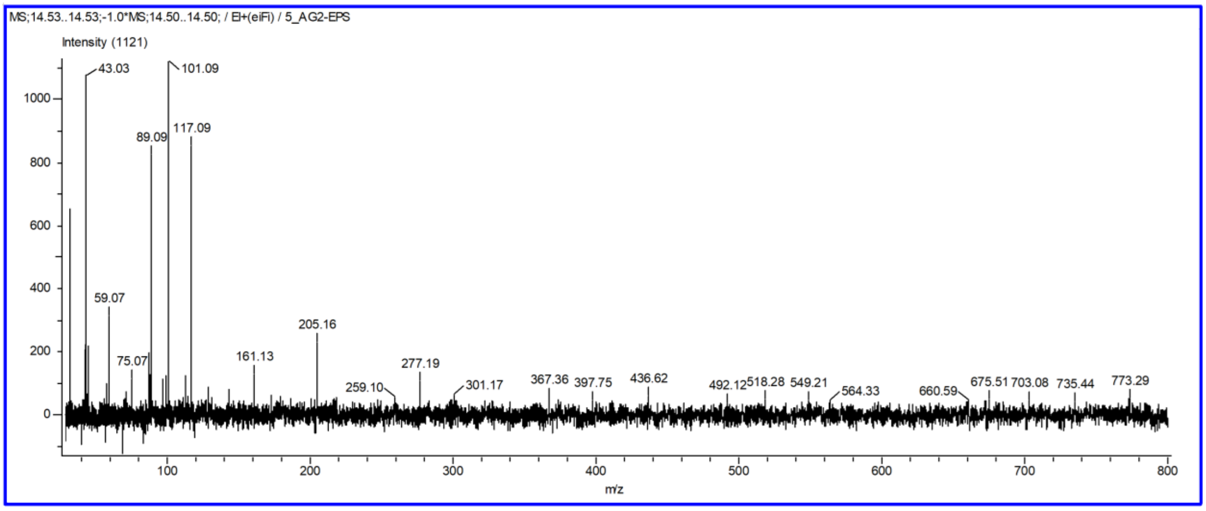
MS;14.53..14.53;-1.0*MS;14.50..14.50; / EI+(eiFi) / 5_AG2-EPS
Intensity (1121)
43.03
101.09
89.09
117.09
59.07
205.16
75.07
161.13
277.19
259.10
301.17
367.36
397.75
436.62
492.12
518.28
549.21
564.33
660.59
675.51
703.08
735.44
773.29
m/z

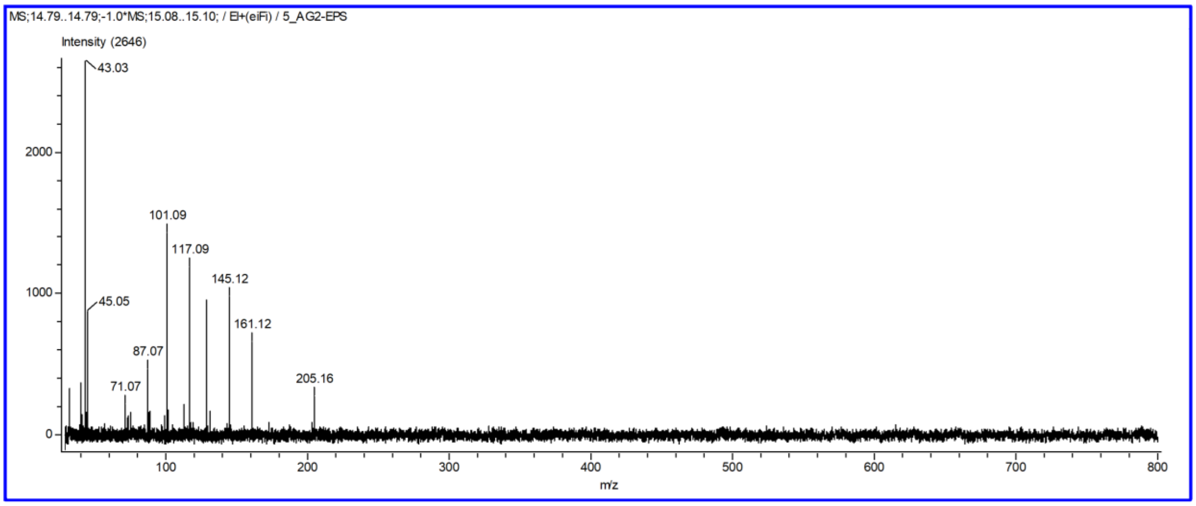
MS;14.79..14.79;-1.0*MS;15.08..15.10; / EI+(eiFi) / 5_AG2-EPS
Intensity (2646)
43.03
101.09
117.09
145.12
45.05
161.12
87.07
71.07
205.16
m/z

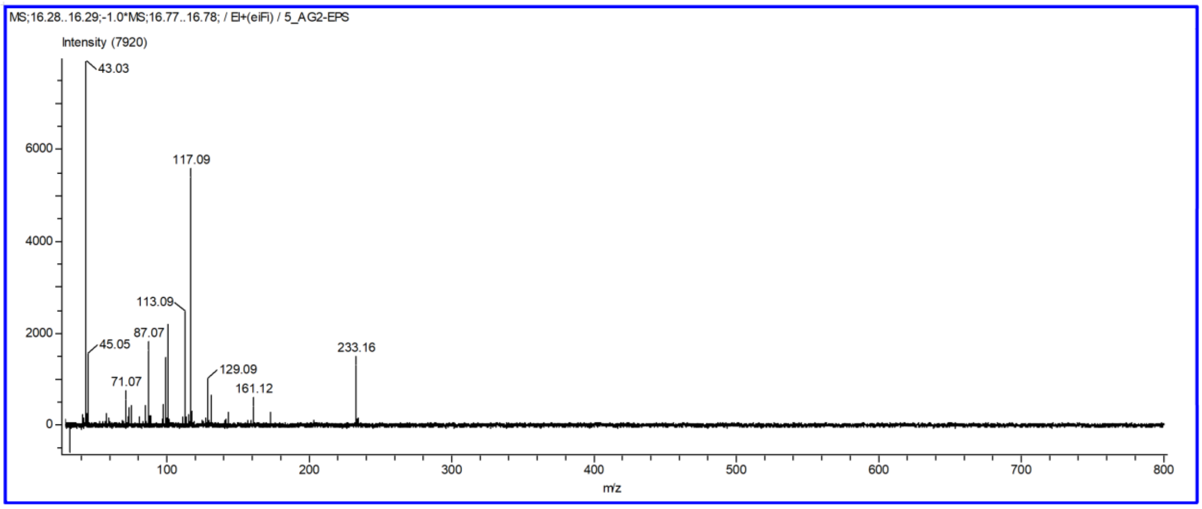
MS;16.28..16.29;-1.0*MS;16.77..16.78; / EI+(eiFi) / 5_AG2-EPS
Intensity (7920)
43.03
117.09
113.09
87.07
45.05
233.16
129.09
71.07
161.12
m/z

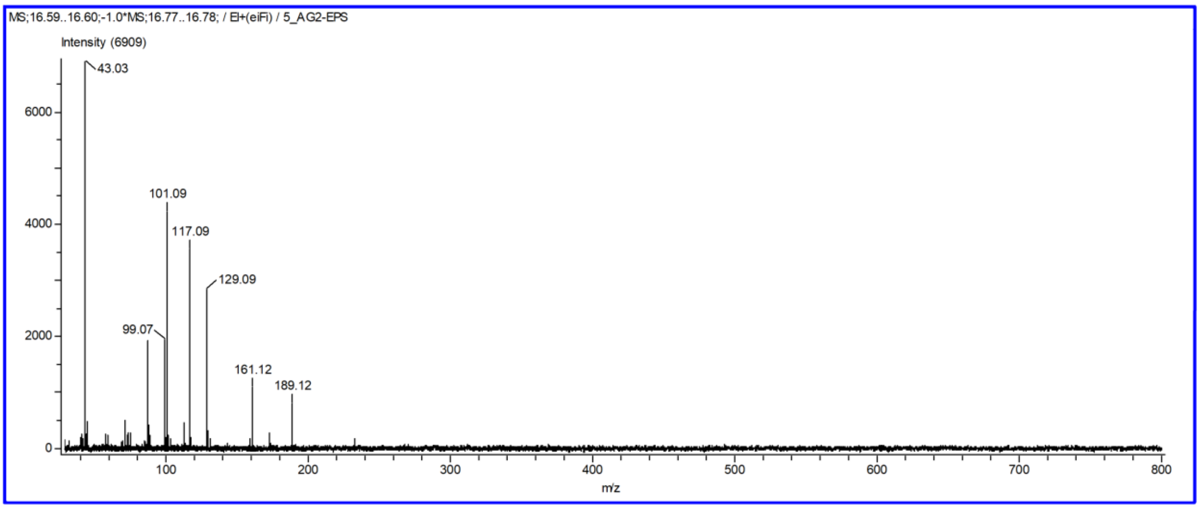
MS;16.59..16.60;-1.0*MS;16.77..16.78; / EI+(eiFi) / 5_AG2-EPS
Intensity (6909)
43.03
101.09
117.09
129.09
99.07
161.12
189.12
m/z

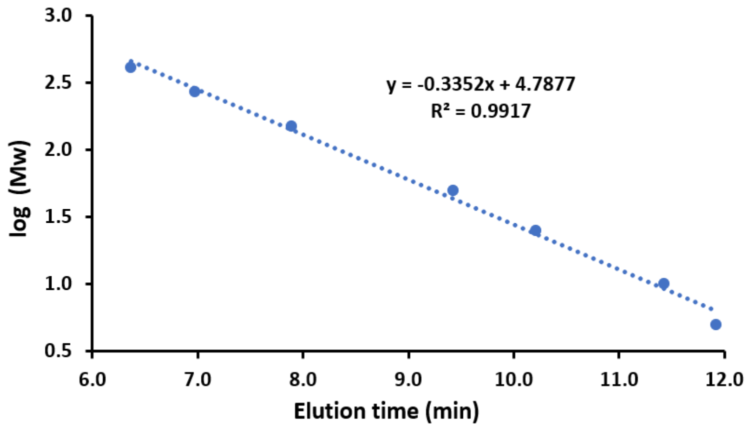

Standard	Mw (kDa)	log Mw	Elution time (min)
S1	5	0.699	11.92
S2	10	1.000	11.43
S3	25	1.398	10.21
S4	50	1.699	9.43
S5	150	2.176	7.89
S6	270	2.431	6.98
S7	410	2.613	6.37

Figure S1. The Dextran calibration curve depicts elution time as a function of varying molecular weights (5, 10, 25, 50, 150, 270, and 410 kDa) using HPLC (2.0 mg/mL in Na_2SO_4 solution of 50 mmol/L, TSK-gel G4000SW column, RI detector, 1.0 mL/min, 30 °C).

(a)

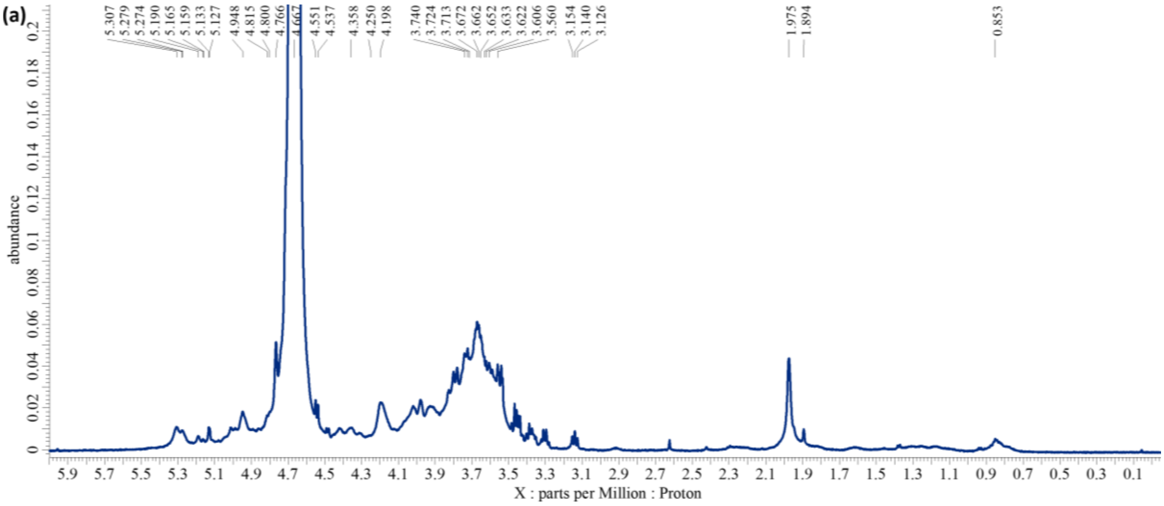
5.307
5.279
5.274
5.190
5.165
5.159
5.133
5.127
4.948
4.815
4.800
4.766
4.667
4.551
4.537
4.358
4.250
4.198
3.740
3.724
3.713
3.672
3.662
3.652
3.633
3.622
3.606
3.560
3.154
3.140
3.126
1.975
1.894
0.853
abundance
X : parts per Million : Proton

(b)

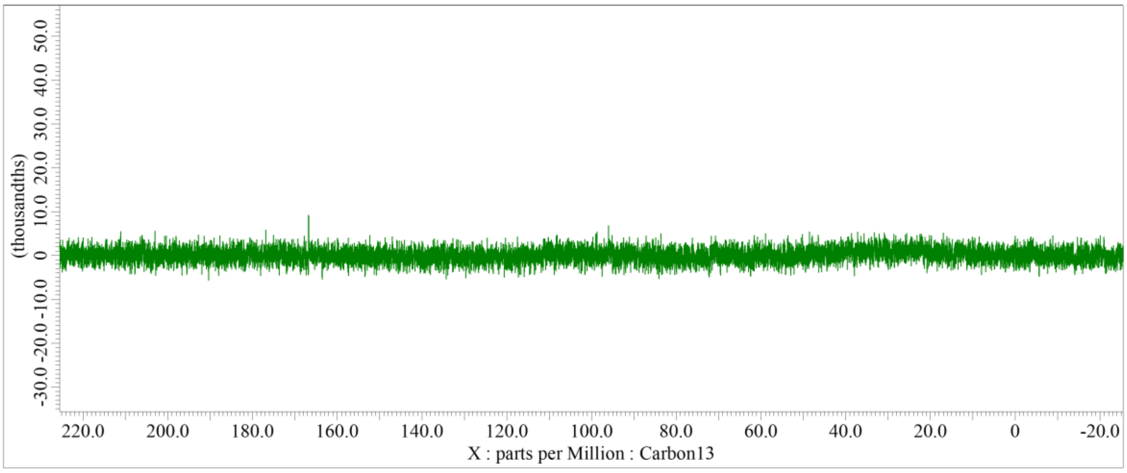
(thousandths)
X : parts per Million : Carbon13

(c)

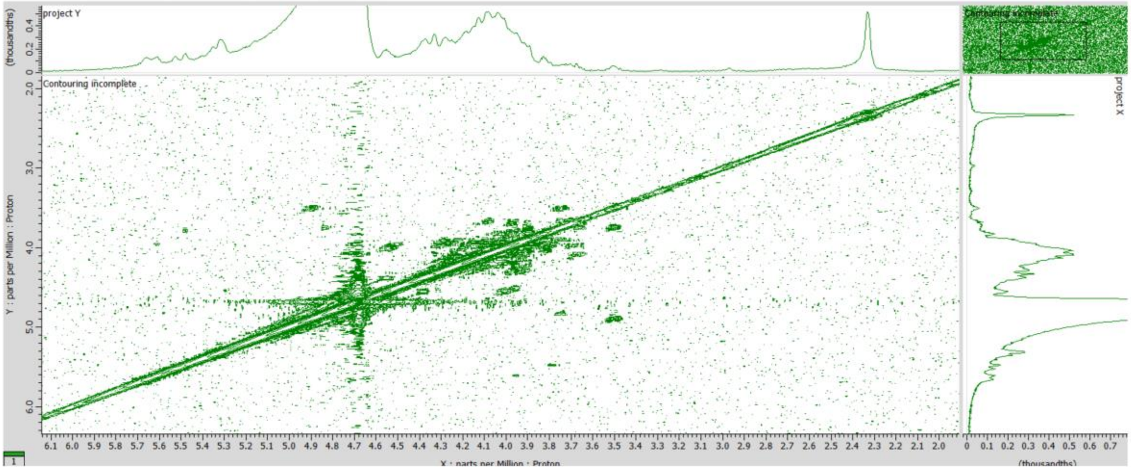
project Y
(thousandths)
Contouring incomplete
Y : parts per Million : Proton
project X

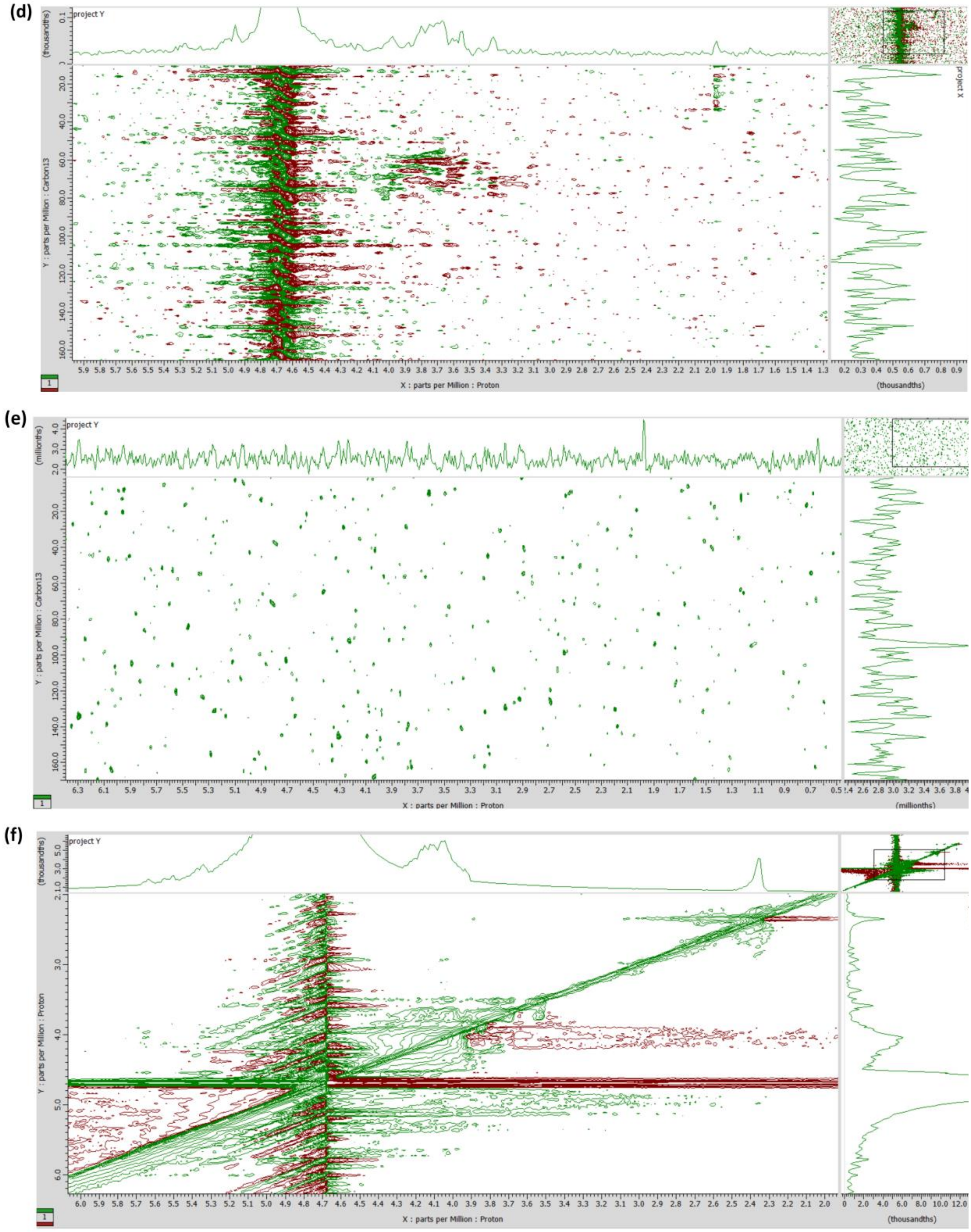

Figure S2. (a) ^{1}H NMR, (b) ^{13}C NMR, (c) COSY, (d) HSQC, (e) HMBC, and (f) TOCSY spectra of the EPS-AG7 produced by *Aspergillus* sp. strain GAD7 (40 mg/mL in D_2O at 298K).

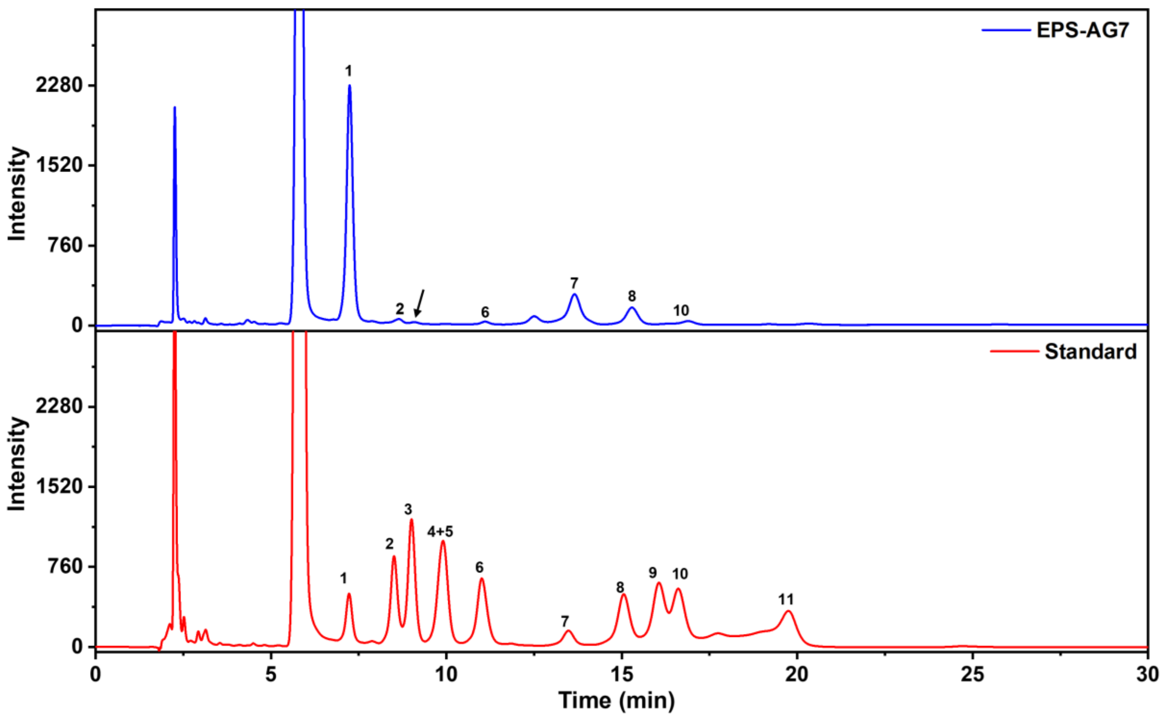

Figure S3. HPLC chromatograms for monosaccharides of the EPS-AG7 produced by *Aspergillus* sp. strain GAD7 compared with monosaccharide standards. Peaks: 1: mannose; 2: lyxose; 3: ribose; 4+5: rhamnose+glucuronic acid; 6: galacturonic acid; 7: glucose; 8: galactose; 9: xylose; 10: arabinose; 11: fucose.

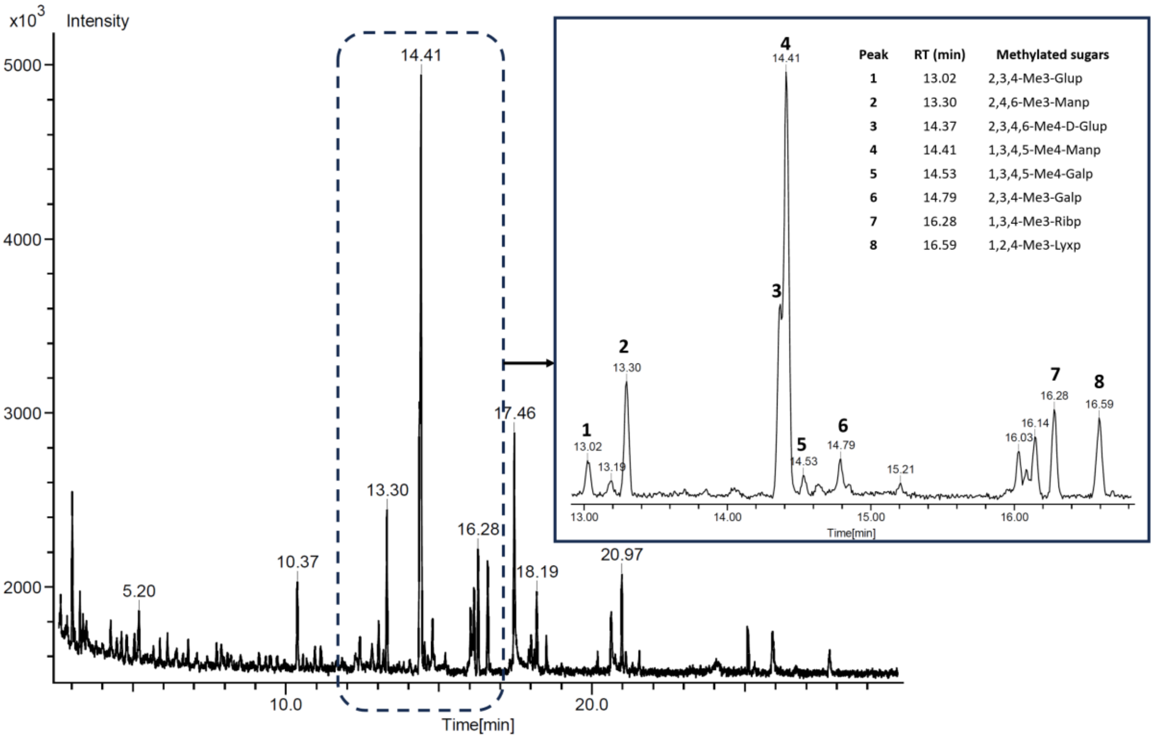

Figure S4. GC chromatogram for monosaccharides of the EPS-AG7 produced by *Aspergillus* sp. strain GAD7.
